# Bridging the digital divide for people with aphasia: a study protocol for codesigning web accessibility tools and guidelines

**DOI:** 10.1136/bmjopen-2025-099273

**Published:** 2025-08-10

**Authors:** Jennifer Lee, Peter Worthy, Ryan Deslandes, Bridget Burton, David A Copland, Phill Jamieson, Kim Barron, Leanne Togher, Kirstine Shrubsole, Ciara Shiggins, Jessica Campbell, Annie Hill, Janet Wiles, S Alexander Haslam, Sarah J Wallace

**Affiliations:** 1 Queensland Aphasia Research Centre, The University of Queensland, Herston, Queensland, Australia; 2 Surgical, Treatment and Rehabilitation Service (STARS) Education and Research Alliance, The University of Queensland and Metro North Health, Herston, Queensland, Australia; 3 School of Health and Rehabilitation Sciences, The University of Queensland, St Lucia, Queensland, Australia; 4 School of Electrical Engineering and Computer Science, The University of Queensland, St Lucia, Queensland, Australia; 5 School of Health Sciences, Faculty of Medicine and Health, The University of Sydney, Sydney, New South Wales, Australia; 6 Centre for Research Excellence in Aphasia Recovery and Rehabilitation, Melbourne, Victoria, Australia; 7 Department of Speech Pathology, La Trobe University, Melbourne, Victoria, Australia; 8 School of Psychology, The University of Queensland, St Lucia, Queensland, Australia

**Keywords:** Digital Technology, Community-Based Participatory Research, STROKE MEDICINE

## Abstract

**ABSTRACT:**

**Introduction:**

Aphasia is a language impairment that affects one-third of people who experience a stroke. Aphasia can impact all facets of language: speaking, understanding, reading and writing. Around 60% of people with aphasia have persistent language impairments 1 year after their stroke, requiring ongoing healthcare and support. In recent years, the internet has become a key resource for the self-management of chronic health conditions. Navigating web content, however, requires language use, and as such, people living with aphasia are more likely to be excluded from digital health and support services. Web Content Accessibility Guidelines exist; however, they do not fully address the unique and diverse needs of people with aphasia, and a significant proportion of websites (over 90%) do not fully adhere to them. This protocol paper describes the first two stages of the *Bridging the Digital Divide* project, which aims to codesign and develop (a) a web-browser extension to re-render webpages to an ‘aphasia-friendly’ (accessible) format, (b) training tools to help users and health professionals customise the web-browser extension and (c) guidelines for developing communication-accessible websites.

**Methods and analysis:**

The research will be conducted using experience-based codesign. In Stage 1a, focus groups will be held with (1) people with aphasia, (2) family members or significant others and (3) health professionals working with people with aphasia. Participants will be asked to share their experiences of accessing (or supporting a person with aphasia to access) healthcare, information and support services on the web. The nominal group technique (NGT) will be used to identify priorities for improving web accessibility for people with aphasia. Focus group data will be analysed using reflexive thematic analysis, and prioritisation data will be analysed using inductive qualitative content analysis. In Stage 1b, eight codesign workshops will be held with representatives of the three key stakeholder groups to iteratively codesign and develop a web-browser extension, training tools and guidelines to support web accessibility.

**Ethics and dissemination:**

Ethical clearance for Stage 1a and Stage 1b of this project has been approved by the University of Queensland Human Research Ethics Committee (Stage 1a approval number: 2023/HE000528, Stage 1b approval number: 2024/HE000721). The outcomes of this research will be disseminated in peer-reviewed journals and presented at national and international conferences. A dissemination and celebration event will be held at the completion of the project.

**STRENGTHS AND LIMITATIONS OF THIS STUDY**

- This study will use experienced-based codesign with people with aphasia, family members or significant others and health professionals to explore factors influencing digital access and to establish priorities for improving accessibility.
- People with lived experience of aphasia will be included in all stages of the project to improve the likelihood that the web-browser extension will be usable and acceptable to the intended population.
- Partnerships have been established with six Australian Government, industry and community organisations to support (1) the development of the web-browser extension, (2) the implementation of the web-browser extension and training package and (3) the dissemination of project outputs and outcomes.
- All participants and codesigners recruited to this study will have poststroke aphasia, and therefore, the project findings and outputs may not be generalisable to people with aphasia from other aetiologies.

## Introduction

 Communication is central to everyday life. It facilitates access to information and connection with others. The importance of communication is recognised in Article 19 of the Universal Declaration of Human Rights,^1^ which states that all people have the right to freedom of expression and the right to seek, receive and impart information. People with communication disability, however, experience many barriers that prevent them from exercising these rights.^2^ One such communication disability is aphasia, a condition that can affect all facets of language (speaking, understanding, reading and writing).^3^ Aphasia results from brain injury, caused by conditions including stroke, tumour, traumatic brain injury, epilepsy and dementia.^3^ Aphasia occurs most commonly following stroke, affecting around one-third of people after stroke.^4^ The degree of impairment and language domains affected by aphasia differs between individuals, and some people will also experience impairments to cognition, including attention, memory, planning, sequencing and troubleshooting.^5^ It is estimated that more than 140 000 Australians live with aphasia.

Recovery from poststroke aphasia can be improved with appropriate and timely speech and language therapy.^6^ Most people with aphasia, however, will have persistent language impairments 1 year after their stroke,^7^ requiring long-term healthcare and support.^8^ Self-management is an effective model of care for the management of chronic health conditions that encourages people to take control and make informed decisions about their healthcare.^9^ The provision of self-management strategies to people with aphasia and their significant others is considered best practice.^10^ Self-management can promote self-efficacy,^11 12^ positively impacting chronic disease management,^12 13^ reducing health service utilisation^14^ and improving quality of life.^15^ For people who have had a stroke, lower self-efficacy is associated with poorer rehabilitation outcomes for mood and well-being, basic and instrumental activities of daily living and mobility.^13^ Thus, using self-management approaches to enhance self-efficacy in people who have had a stroke may be key to maximising recovery outcomes.

For people with aphasia, access and support to engage with technology are important components of self-management.^16 17^ The shift from in-person interactions to digital engagement can improve ease of access to services and reduce burden on service providers.^18^ However, language and cognitive skills are needed to access online services and successfully navigate the digital world. As such, people living with aphasia are at greater risk of digital exclusion, which can create a digital divide.^19 20^ The digital divide is a term used to describe the gap between people who do and do not have the resources, skills or confidence to use modern digital technology.^21^ Socioeconomic status, age, education, employment, location and disability are all factors that influence whether an individual can bridge the digital divide.^22^ While rates of digital inclusion in Australia have steadily improved since 2020, one in four people still experience digital exclusion.^23^ The groups most impacted include First Nations peoples, those with low income or are unemployed, people living in rural and remote areas, those aged over 75 and people who live with disability.^23^ van Dijk and Hacker^21^ identified four main barriers to digital access: (1) lack of elementary digital experience, (2) lack of access to a computer and reliable internet connection, (3) lack of digital skills caused by poor or inappropriate usability and limited education or social support and (4) lack of usage opportunities. While the impact of disability is not explicitly highlighted in these four barriers, recent studies have concluded that having a disability exacerbates all of these challenges.^19 20 24 25^

Compared with other disabilities, a larger proportion of people with communication and language disabilities report not feeling included in digital society.^24^ Some situations where difficulties can arise include booking a medical appointment, searching for information, navigating online information, trying to understand that information and management of passwords and other internet security features.^24^ Information-seeking on the web can be challenging due to word retrieval deficits, rejected search queries from spelling errors and memory and concentration lapses.^26^ Assessing the reliability of search results returned from a search query can also be difficult for people with communication or language disabilities.^27 28^ Consequently, people with communication disabilities, such as aphasia, are less likely to use and have greater avoidance of digital health services.^25^ Exclusion from digital services can have direct and indirect health consequences.^22^ Direct health consequences include not being able to book a medical appointment or seek health information, including secondary stroke prevention material. Indirect health consequences include inequitable access to employment and education opportunities and increased social isolation, leading to negative well-being. In recent years, guidelines for inclusive website design have been developed to promote digital access for people with disability. The Web Content Accessibility Guidelines (WCAG) developed by the World Wide Web Consortium (W3C) provide guidelines for creating accessible websites.^29^ Historically, they have focused primarily on sensory and physical disabilities, with less emphasis on cognitive and learning disabilities.^30 31^ The W3C’s Cognitive and Learning Disabilities Task Force was established to address these limitations and to develop guidelines and resources that include the needs of people with cognitive and learning disabilities specifically,^32^ including people with language impairments. However, the needs and preferences of people with aphasia are unique and diverse and have not been fully addressed by the current guidelines. Accessibility audits of selected public health websites have demonstrated that up to 91% of home pages of public health websites are not compliant with the WCAG 2.0 Level AA accessibility standards.^33 34^ People with aphasia and communication disability are at risk of digital exclusion. New solutions, guidelines and training are needed to support their digital access.

This publication presents a protocol for the codesign and development of technology to improve web access for people with poststroke aphasia. While the inclusion of people with aphasia in codesign is feasible and well-reported,^35^,^38^ the use of experience-based codesign (EBCD)^39^ to design and develop a web-browser extension for people with aphasia is novel. The combination of EBCD and human-centred design^40^ is also a novel approach. This approach may form a useful methodology for people using human-centred design in technology development to focus on lived experiences and significant moments that shape a user’s experience. This protocol is therefore presented with the aims of (1) describing important adaptations and considerations that may benefit others working in similar contexts; (2) informing researchers of our ongoing work, potentially reducing duplication of effort and facilitating collaboration and (3) enhancing methodological transparency and replication or adaptation of our approach.

## Study aims

The overarching aim of the *Bridging the Digital Divide* project is to codesign and develop a novel intervention that will support web access for people with aphasia. The intervention will consist of three components:

1. A communication accessibility web-browser extension—a novel and customisable software that will re-render website content to meet individual communication support needs. The browser extension will also support information access through a recommender system that will generate suggestions for health- and support-related websites based on individual needs and preferences.
2. A decision-making tool and training package that can be used by health professionals to customise the browser extension and train people with aphasia to use it.
3. Guidelines for communication-accessible website design to increase awareness and knowledge of communication-access needs.

**Figure 1:**
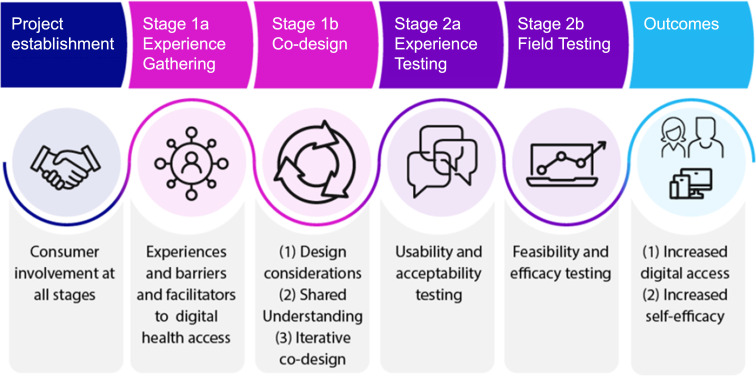
*Bridging the Digital Divide* project overview.

The *Bridging the Digital Divide* project consists of multiple stages (figure 1). This publication presents the protocol for Stage 1a (Experience Gathering) and Stage 1b (Codesign). Stage 1a will involve conducting focus groups to develop a shared understanding of stakeholder experiences of using, and supporting use of, the web with aphasia. The outcomes of Stage 1 a will provide the foundation for Stage 1b, a series of codesign workshops. The aims of Stage 1a and Stage 1b are outlined in table 1.

**Table 1:** Aims for Stages 1a and 1b of the *Bridging the Digital Divide* project

Stage of the project	Aims
Stage 1a: Experience Gathering	Understand experiences of people with aphasia in using the web, with focus on accessing online healthcare, information and support services. Identify how family members or significant others and health professionals support a person with aphasia to use the web. Identify touchpoints and unmet needs and determine key priorities for improving web accessibility for people with aphasia for self-management of health.
Stage 1b: Codesign	Refine and expand the preliminary design framework through experiences and priorities identified from Stage 1a. Iteratively codesign the web-browser extension with key stakeholders, including development, testing and evaluation of web-browser extension prototypes. Identify implications and considerations arising from the codesign and technology development that will have broader applications for different technologies and contexts (eg, development of app-based technology).

## Methods and analysis

### Patient and public involvement

People with lived experience of poststroke aphasia will be involved across all stages of this project. Authors PJ and KB have been appointed as lived-experience chief investigators within the research team. A consumer advisory group comprising four people with poststroke aphasia and four unrelated family members of people with poststroke aphasia has been established. This group will guide research procedures and review project materials (eg, participant recruitment flyer, participant information and consent forms, focus group guide). The consumer advisory group will be paid an honorarium in alignment with the Health Consumers Queensland standards.

Partnerships have been established with six Government, industry and community organisations: (1) Australian Aphasia Association (a not-for-profit organisation and registered charity which advocates for people living with aphasia in Australia), (2) Australian Disability Network (a national peak body helping employers build capacity to include people with disability as employees and customers), (3) Centre for Accessibility Australia (a not-for-profit organisation that promotes digital accessibility in Australia), (4) National Disability Insurance Agency (an independent statutory agency which administers the National Disability Insurance Scheme and provides support for Australians with significant and permanent disability), (5) Services Australia (an executive agency of the Australian government who are responsible for delivering accessible social services and payments to eligible Australian citizens) and (6) the Australian Stroke Foundation (a national charity organisation that partners with the community to prevent stroke, save lives and enhance recovery). A steering committee has been established with representatives from each partner organisation. Partner organisations will guide and support: the development of the browser extension, recruitment, implementation of the browser extension training package and communication-accessible web guidelines and dissemination of findings. The Centre for Accessibility Australia will conduct accessibility audits of webpages re-rendered by the browser extension to assess whether they meet accessibility standards.

### Design

The *Bridging the Digital Divide* project will combine EBCD^39^ and human-centred design approaches.^40^ The EBCD process consists of two stages (Stage 1 and Stage 2), each with two substages (eg, Stage 1a and Stage 1b; figure 1). Stage 1 will use participatory methods to understand experiences, unmet needs and priorities for key stakeholders and then collaboratively codesign a solution using design thinking and learning theory.^41 42^ In EBCD, learning theory emphasises the importance of mutual learning through collaboration with key service users, service providers, designers and researchers to identify key touchpoints (ie, moments of significance that shape a user’s experience) and areas for improvement.^39 42^ A growing number of healthcare service improvements have used the EBCD approach.^41^,^43^ Human-centred design is a design methodology that focuses on the user’s needs, experiences and knowledge in the design process^44^ and is commonly used in the design of a technological system. The goal of human-centred design is to create a product or solution that addresses the unique needs and challenges of the population in mind. Using the combination of EBCD and human-centred design approaches ensures that the needs, experiences and insights of the people who will ultimately use a service or technology are embedded in the design process.^44 45^ Stage 2 of the project comprises experience and field testing to evaluate whether the web-browser extension and training tools are usable, acceptable and effective in improving self-efficacy in people with aphasia for accessing self-management services online. Both approaches allow for flexibility in making modifications to support the involvement of populations with specific needs^46 47^ and have been successfully used with people living with aphasia^48^ and to develop mobile applications to support stroke rehabilitation.^49^

This paper details the protocol for Stage 1a (Experience Gathering) and Stage 1b (Codesign) of the EBCD process. The outcomes from Stage 1a will inform the preliminary design framework for Stage 1b which will involve a series of eight codesign workshops with researchers, software developers, people with aphasia, family members or significant others and health professionals working with people with aphasia to iteratively develop, trial and refine the web-browser extension. The project commenced in June 2023 and the anticipated completion date is December 2025.

### Participants and codesigners

At all stages of the *Bridging the Digital Divide* project, input will be sought from three key stakeholder groups: (1) people living with poststroke aphasia, (2) family members or significant others and (3) health professionals working with people with poststroke aphasia. For Stage 1a, individuals will be recruited as participants, meaning they will be contributing data to the project, and consent will be obtained. In Stage 1b, individuals will have the role of a codesigner. Codesigners will be involved in ideation, decision-making and feedback provision throughout the codesign process. In this project, it is possible for participants who take part in Stage 1a to be involved as codesigners in Stage 1b. Participants from Stage 1a will be asked at the point of consent whether they agree to be contacted for future research studies associated with the project. Those who agree to be contacted will be sent an email invitation at the commencement of Stage 1b.

The eligibility criteria for Stage 1a and Stage 1b are identical. All participants must be over 18 years of age, reside in Australia, have normal or corrected-to-normal vision and hearing, have adequate proficiency in English to contribute to group discussions and be able to provide informed consent. Individuals with aphasia are eligible to participate if they are at least 3 months poststroke and can participate in group sessions with communication support, if required. The exclusion criteria for individuals with aphasia will be severe cognitive impairment, people who cannot provide informed consent, presence of other neurodegenerative conditions such as dementia or Parkinson’s and aphasia aetiologies other than stroke. Health professionals may include speech pathologists or other health disciplines who have experience working clinically with people with poststroke aphasia. The sampling criteria for each participant group are described in the next section.

### Sampling

We aim to include 10 members from each stakeholder group in Stage 1a and Stage 1b, as recommended by the Agency for Clinical Innovation (https://aci.health.nsw.gov.au/) based on previous EBCD studies. This will enable a range of perspectives to be captured. For individuals with aphasia, sampling will consider age, severity of aphasia as indicated by the Aphasia Severity Rating Scale,^50^ time poststroke and gender identity. A conversation sample from the initial meeting with the researcher will be used to evaluate language ability and aphasia severity. This will be independently reviewed by two members of the research team who hold speech pathology qualifications.

Family members or significant others will be recruited via convenience sampling according to the inclusion criteria outlined above. These individuals may have a relationship with a participant or codesigner with aphasia enrolled in the study, but this is not a requirement. Health professionals will be recruited using purposive sampling with disciplinary background (eg, speech pathologists, occupational therapists), clinical setting (eg, acute rehabilitation, outpatient rehabilitation, community) and years of experience working with people with aphasia taken into consideration.

#### Supported communication with people with aphasia

All researchers and software developers involved in this project have received formal communication partner training with a qualified speech pathologist. Researchers can provide one-to-one support for people with aphasia during focus groups and in the codesign workshops to facilitate discussion. Communication resources, such as Talking Mats, and communication support techniques (eg, reduced speech rate, use of written key words, drawings and hand gestures, use of fixed-choice questions when appropriate) will be used in all discussions. Individuals with aphasia may also have a support person accompany them to meetings and workshops to provide support.

### Recruitment and informed consent

All documentation intended for people with aphasia (ie, recruitment advertisement, participant information sheet and consent forms) will be developed in an aphasia-friendly format^51^ and reviewed by authors KB and PJ and the consumer advisory group. Informed consent will be obtained from all individuals prior to commencing any research activities. This may include witnessed oral consent for people with aphasia. In codesign, individuals may take part in multiple stages of the project, if desired. Separate consent will be sought at each stage of the project, so individuals will have the opportunity to consider their involvement at each stage. Consent will also be sought from participants for the use of their words or images in the development of the touchpoint materials in Stage 1a. Touchpoint materials may include figures, presentations and/or videos that highlight the critical moments in participants’ experiences (see Anemaat *et al*^52^ for an example of a touchpoint film). A participant’s decision of whether they consent to having their words or images used in touchpoint materials will not impact their opportunities to participate in subsequent stages of this project.

For recruitment in Stage 1a, a brief advertisement with the project aims, eligibility, what participation entails and contact information will be sent via email to aphasia networks within Australia (eg, Australian Aphasia Association, Queensland Aphasia Research Centre). These organisations will circulate the advertisement to their members via email or through their newsletter. For health professionals, the advertisement will be circulated via email or word-of-mouth through professional networks and newsletters. Individuals who are interested in participating may fill out an expression of interest form or contact the researchers for more information. For recruitment in Stage 1b, participants who completed the Stage 1a focus groups and expressed an interest in being involved in subsequent stages of the project will be emailed a participant information sheet for Stage 1b. If an insufficient number of participants from Stage 1a wish to take part in Stage 1b, a brief advertisement with the project aims, eligibility, roles and responsibilities and contact information will be circulated to the networks mentioned above.

#### People with aphasia

Individuals with aphasia who express interest in Stage 1a and/or Stage 1b will be emailed the relevant participant information sheet. A researcher with training in supported communication techniques will schedule a teleconference meeting with the individual with aphasia to explain their involvement in the study and to obtain consent, where applicable. A family member or support person may also attend this meeting to provide support. Once the study information has been explained to the potential participant, the researcher will verify that they have understood the requirements of their participation/involvement by asking the participant to respond ‘true’ or ‘false’ to three statements about the study.^53^ Participants will also complete a demographics questionnaire with information in line with the DESCRIBE reporting standards^54^ and a questionnaire about their prestroke and current experience with technology use.

#### Family members or significant others

Family members or significant others who express interest in Stage 1a and/or Stage 1b will be emailed the relevant participant information sheet. Individuals may express interest in the study with a family member who has aphasia; however, this is not a requirement. If a family member would like to enrol in the study with a participant with aphasia, they may attend a teleconference meeting together to go through the consenting procedures outlined above. If a family member would like to enrol as an individual, they will be given the opportunity to discuss their involvement in the study with a member of the research team over the phone or via Zoom. If they wish to participate, they will be asked to return a signed consent form via email or post. Participants and codesigners will also complete a basic demographics questionnaire.

#### Health professionals

Health professionals who express interest in Stage 1a and/or Stage 1b will be emailed the relevant participant information sheet. They will also be asked to provide some basic information about their work experience, which will be used to select participants with diverse experiences to maximise sampling variation. Selected individuals will then be asked to return a signed consent form via email or post if they wish to participate.

#### Reimbursement

All participants and codesigners will be reimbursed for their time and travel. Individuals with aphasia and family members or significant others will be reimbursed according to the rates recommended by Health Consumers Queensland at the time of their participation. Health professionals will be reimbursed at the rate of A$60 per hour.

### Procedure

#### Stage 1a: Experience Gathering

##### Experiences of accessing and using healthcare, information and support services on the web

Focus groups will be held with (1) people living with aphasia, (2) family members or significant others and (3) health professionals either in-person at the University of Queensland Herston campus or online via Zoom. Separate focus groups will be held for each stakeholder group with no more than five participants allocated to each group. Limiting the number of participants will increase the likelihood that everyone has sufficient opportunity to contribute to the discussion. The collaborative nature of focus groups allows for diverse perspectives to be heard and encourages discussion between participants.^55^ Online focus groups via Zoom are an acceptable and feasible way for people with aphasia to engage with technology and participate in research.^56^ Optional training sessions for using Zoom and its functions can be provided to participants who require, or wish to have, additional support. Discussions in the focus groups will be facilitated by one researcher who will present the questions to the participants. For the focus groups with people with aphasia, up to three members of the research team with training in supported communication techniques will be present to support communication and engagement in focus group activities. All focus groups will be video and audio recorded for data analysis. All participants will be asked to provide consent for the recording during the initial consenting procedures. The researchers will confirm consent from all participants prior to starting the recording at each meeting. If a participant does not wish to be recorded, they may opt to attend another focus group if available or withdraw from the study.

A focus group discussion guide (see online supplemental materials) will be used to ensure that the key questions are consistently captured across focus groups. The discussion guide consists of open-ended questions with follow-up questions to facilitate discussion during the focus groups. Participants will be prompted to:

1. Discuss their experiences of using, or supporting someone with aphasia in using, the web to access healthcare, information and support services.
2. Share one positive and one negative experience of using the web for this purpose.
3. Suggest ways in which their experience of using, or providing support for using, the web could be improved.

Participants with aphasia will also be asked to share the level and type of support they receive when using the web. Previous research has demonstrated that while some people with aphasia were able to independently navigate the web, it was also common for people to use the internet via a proxy (ie, someone else who completes tasks on their behalf).^20^ This will enable us to capture the range of digital independence across different web-based tasks and contexts and consider the level of support that can be provided through the browser extension.

Supported communication techniques will be used during focus groups to enhance accessibility for people living with aphasia. These may include multimodal presentation of information, providing meeting agenda and focus group questions with accompanying audio recording prior to the focus group to allow time to process the information and/or generate responses, and allowing additional time for comprehension and response generation within the group. The focus group discussion guide has been reviewed with authors KB and PJ and the consumer advisory group to check that the questions are relevant and appropriately worded to optimise understanding.

Participants with aphasia may choose to be accompanied by a support person to provide additional communication support. The primary role of the support person will be to help the participant with aphasia understand the focus group questions and to express their answers. However, we acknowledge that there may be instances where the support person may speak on behalf of the person with aphasia, thus acting as a communication proxy, or share their own perspectives. If this arises, the facilitator will verify with the participant with aphasia that the information accurately reflects their experiences.

##### Generation of essential features to support web accessibility

The NGT^57^ will be used to identify factors that would help an individual with aphasia to access healthcare, information and support services online, or to improve the provision of support for accessing these services online. The NGT is a structured group decision-making technique that encourages contributions from all participants and is an effective way of gaining group consensus. This technique has successfully been used to facilitate discussion with people with poststroke aphasia.^48 56^ Participants will be asked to generate ideas in response to the nominal question “What factors would support/help you in using the internet to access healthcare, information and support services?” Each response will be recorded on a Miro board (www.miro.com) as they are shared. The ideas shared may be discussed further, if necessary, to clarify the idea and to ensure that the intended meaning of the participant was captured. Following this, similar ideas will be grouped together.

##### Ranking essential features

Following the generation of ideas, each participant will be asked to individually select five ideas from the Miro board that they consider to be most important for improving web accessibility. They will then be asked to rank their selected ideas from 1 (least important) to 5 (most important) and assign each idea a relative importance score between 1 and 100. The most important idea will be given the highest score, and the combined total of all five scores must equal 100 points. Participants will be given approximately 10 min to complete this activity individually. Research team members can provide one-to-one support to participants, if required. Once all participants have completed their ranking and assignment of points, the facilitator will ask each participant to share their selected ideas and scores and will record this on the Miro board.

##### Data analysis

Discussions from the focus groups will be transcribed and analysed using reflexive thematic analysis.^58 59^ Key touchpoints that arise from the focus group discussions will be used to create resources (eg, journey map) to highlight experiences that are of emotional significance. These resources will be presented to the codesigners in Stage 1b and used as the foundation for designing and developing the web-browser extension.

Rankings of the key priorities identified for supporting web accessibility will be calculated based on the sum of the scores, the ranked priority by score, the relative importance, the ranked priority by per cent, frequency of voting and the ranked priority by score and frequency.^50^ Qualitative content analysis will be used to combine and compare priorities across groups.^60^ Themed priorities will be used to develop problem statements for review and prioritisation during the next stages of the project.

### Stage 1b: Codesign

#### Codesign workshops

Following the identification of key priorities for improving web accessibility from Stage 1a, eight codesign workshops will be held with (1) people living with aphasia, (2) family members or significant others and (3) health professionals who work clinically with people with aphasia. These workshops will involve an iterative codesign process, occurring in parallel with the development and prototype testing of the browser extension.^61^ Each workshop will be up to 2 hours in duration and spaced approximately 2 weeks apart. It is anticipated that codesigners will contribute up to 18 hours of their time during the codesign process. This includes time spent attending the codesign workshops and any activities completed between workshops.

The workshops will be facilitated by members of the research team who have experience in working with and codesigning technology for people with aphasia (SJW, PW, RD, JL). SJW is a speech pathology with extensive experience in codesigning services and technology with people with communication disability. PW is an interaction designer with experience in codesigning and developing technology for a wide range of populations, including people with dementia and aphasia. RD is a software developer with experience in codesigning and developing technology for people with dementia and aphasia. JL has a background in cognitive psychology and experience in working with people with aphasia. Two research assistants with lived experience of aphasia (KB, PJ) will provide guidance for the codesign process to ensure that the workshop activities will be communicatively accessible.

Codesigners will receive a welcome pack with materials that may be useful during the codesign workshops. This will include a design journal, stationery and voting cards. Codesigners may also use their preferred method of journaling during the codesign sessions (eg, electronic design journal, voice memos). The voting cards may be used for getting consensus for features or designs to be included in the web-browser extension and include descriptors ‘Must have’, ‘Should have’, ‘Maybe’, ‘Shouldn’t have’, ‘Definitely not’. The voting cards are modified based on the MoSCoW Prioritisation system.^62^

The initial codesign workshops (workshops 1–2) will provide codesigners with information about their roles and responsibilities, use of respectful language in workshops, confidentiality obligations and rights to intellectual property and the aims, process and expectations of codesign. Researchers facilitating the codesign workshops will identify individual accessibility needs during the initial workshops so that these can be accommodated for during the workshops. An overview of the broader research project, outcomes from the Stage 1a focus groups and knowledge about existing accessibility software and assistive technology will also be shared in the initial codesign workshops. Workshops 3–7 will be dedicated to generating ideas for designing and creating the browser extension, reviewing existing browser extensions, testing prototypes of the new browser extension, identifying problems that people may experience and developing an understanding of the information that is collected from prototype testing. This may include reviewing and discussing the accessibility of existing web interfaces and development of design mock-ups using Miro to illustrate the user interface and interaction. Codesigners will be encouraged to actively contribute to the design of the user interface through collaboration on a shared Miro board or drawing in their design journals and sharing this with the researchers.

During the codesign workshops, there may be discussions around codesigners’ experiences with using existing technology including issues encountered and possible solutions to those issues, feedback about a concept for a technology or an existing technology, issues about how a technology should work for a user and complete codesign activities including workbooks and feedback. There will also be discussions around security and storage of personal information and data while using the browser extension, as some features (eg, the recommender system) may require access to a user’s browsing data to provide suggestions. All codesign workshops and discussions will be video and audio recorded. Codesigners will be able to access resources and materials from the codesign web page created for the project, including a recording of the workshop content, after each workshop.

Codesigners will also be invited and encouraged to participate in activities between the codesign workshops to further engage them with the codesign process. These optional activities may include communication with other codesigners and the research team via email or instant messaging platforms and maintaining a design journal.

#### Prototype testing of the browser extension

The development of the web-browser extension will be led by PW (interaction designer) and RD (software developer) who both have experience in codesigning and developing technology with people with aphasia. This process will also be guided by KB and PJ who have backgrounds in user experience design and customer experience, respectively. For the purposes of this study, the browser extension will be codesigned and developed for use on a desktop or laptop computer. The browser extension prototypes will be developed and evaluated simultaneously as a part of the iterative design process in Stage 1b. Codesign testers may be asked to participate in a design walkthrough and share their feedback in a semistructured interview, complete the System Usability Scale^63 64^ and rate the web-browser extension prototype against usability heuristics.^61^ For the evaluations of a web-browser extension prototype, participants may be asked to share (1) their experiences with using or customising the prototype, (2) features they liked or disliked about the prototype, (3) any issues they encountered while using the prototype, (4) the level of support required to use or customise the prototype and (5) any additional features they think would be useful. It is anticipated that a total of 3–5 evaluations or testing activities will be conducted throughout the codesign process.

Methods for analysis from the prototype testing will depend on the nature of the data. If both qualitative and quantitative data are used to explore a particular need or experience, the approach adopted will either be Explanatory Sequential Design or Exploratory Sequential Design^65^ depending on the stage in the codesign process and the design issue that is being explored.

#### Next steps: experience testing

Following Stage 1b (Codesign), a series of usability and feasibility testing will be conducted to ensure that the browser extension and training guide can be used as intended by people with aphasia, family members or significant others and health professionals, and to determine whether people are willing to use it. A 4-week field test will examine whether the browser extension is feasible for use in a real-world environment and whether it improves self-efficacy for people with aphasia. Research protocols specific to Stages 2a and 2b (figure 1) will be developed as the project progresses.

#### Review and celebration event

As the final stage of the EBCD process, all participants, codesigners, consumer advisory group members, steering committee members and representatives from partner organisations will be invited to attend a feedback and celebration event at the completion of the project. The outcomes from the project and future directions will be shared at this event.

### Ethics and dissemination

Ethical clearance for Stage 1a and Stage 1b of this project has been approved by the University of Queensland Human Research Ethics Committee (Stage 1a approval number: 2023/HE000528, Stage 1b approval number: 2024/HE000721). Any modifications to the research protocol and documentation will be submitted to the Ethics Committee for review prior to administration.

Informed consent will be obtained from all participants and codesigners prior to commencing any research activities. For people with aphasia, consent will be obtained via videoconferencing. This meeting will be recorded as a means of documenting the consent process. Family members, significant others and health professionals who consent to participating may return their consent to the researchers by email or post. Consent for video and audio recording will be confirmed at the start of each meeting, focus group and/or codesign workshop by monitoring participants’ responses (eg, say yes, nodding, thumbs up). Additional consent will be sought if the researchers wish to use the words, audio or image of participants for research dissemination or in any media presentations. Any participant or codesigner who shows signs of discomfort or distress will be reassured and given the opportunity to withdraw from the study or return to participate at another time.

For all focus groups and codesign workshops, meeting agendas will be circulated to participants and codesigners prior to the event. If feedback is being sought from participants and codesigners, the questions will also be provided ahead of time to allow for the opportunity to consider responses prior to the meeting.

The outcomes of this research will be disseminated in peer-reviewed journals and presented at national and international conferences. A dissemination and celebration event with all individuals involved in the project will be held at the completion of the project.

## Summary

This paper has described the protocol for the first two stages of the EBCD process for the *Bridging the Digital Divide* project; Stage 1a will involve understanding experiences of accessing healthcare, information and support services online for people with aphasia, and experiences of providing support from family members or significant others, and health professionals, and Stage 1b will involve codesigning and developing the web-browser extension. The anticipated outcomes from Stage 1a are a shared understanding of (1) experiences of accessing healthcare, information and support services online from people with aphasia, and experiences from family members, significant others and health professionals for providing support, (2) barriers and facilitators influencing web accessibility and (3) key priorities for improving web accessibility for people living with aphasia. The outcome of Stage 1b is a prototype of the web-browser extension as the result of eight codesign workshops involving iterative design, development and testing.

### Potential impact of research outcomes

The prevalence of people living with stroke and aphasia is rising due to the ageing population.^66 67^ A communication impairment can impact a person with aphasia’s experience with accessing appropriate and timely healthcare and information, social participation, mental well-being and quality of life. With the increasing shift to digital health and support services, it is imperative that these services are inclusive and can accommodate the needs of people with communication disability. The rapid growth of technology can be difficult for some users to grasp, particularly if they do not have the skills and confidence to adopt new technologies into their daily lives.^68^

The current research is strengthened by the involvement of people living with aphasia, family members or significant others, and health professionals throughout the whole design process. By considering the knowledge and experiences of end-users, the EBCD and human-centred design approaches will enhance the likelihood that the needs of end-users are addressed.^44^ Furthermore, this may also increase the prospects that the web-browser extension and training tools will be accepted and can be conveniently integrated into everyday life. As aphasia affects individuals in diverse ways, the development of a customisable web-browser extension will enable individual needs and preferences to be met. The foreseeable limitations in this project include the exclusion of people with aphasia caused by non-stroke aetiologies and that the proposed browser extension will only be compatible with desktop and laptop computers. This may restrict usage for users who prefer to use phone or tablet devices but is a consideration for future research.

It is anticipated that the outcomes from this project will empower people living with aphasia and their family members or significant others to manage their own healthcare and recovery. The training guides will also allow health professionals to build greater confidence in supporting a person with aphasia to access information, healthcare and support services online. A new set of guidelines for communication-accessible websites that has been developed with people with aphasia will improve knowledge and awareness of the web accessibility needs of people with communication disability.

## Supplementary material

10.1136/bmjopen-2025-099273 online supplemental file 1
