# Supplementary material for: Bridging the digital divide for people with aphasia: a study protocol for codesigning web accessibility tools and guidelines
Source: BMJ Open. 2025 Aug 10;15(8):e099273. doi: 10.1136/bmjopen-2025-099273 (PMC12336571; doi:10.1136/bmjopen-2025-099273)
Supplement: online supplemental file 1 [file bmjopen-15-8-s001.pdf]

THE UNIVERSITY OF QUEENSLAND
AUSTRALIA
CREATE CHANGE

Bridging the Digital Divide Project – Focus Group Guides & Demographic Data

Focus Group Guide: Person with Aphasia

Q1. Can you tell me about your experience(s) of using the internet to access health care, information, and support, since your stroke?

Prompts:

i. Think back to the last time that you used the Internet to access health care information or support. Tell me about that experience.
    a. Which site was it?
    b. Why were you going to that site?
    c. What happened when you tried to use the site?
    d. What made the site easy/hard to use?
    e. How often would you need to use that website?
    f. How did that make you feel?

ii. What has been your experience of reading written information on the Internet?
    a. What helps you understand written information and what makes it difficult? (prompts: font size, font, spacing, bolding of key words, headings, white space, images, accompanying audio)
    b. Does bolding of key words help? Which words are key to helping you understand a sentence?

iii. What level of support have you needed to use the Internet?
    a. Who has helped provide this support?
    b. Have you accessed external supports to use the Internet (e.g. from a therapist, support network/group)?

iv. Problem-solving & trouble-shooting on the Internet:
    a. Have there been times where you've had challenges knowing what to search for?
    b. What do you do when you can't think of the word you want to search for?
    c. What do you do if there is a word you cannot recognise?

v. Accessible Websites (Aphasia-friendly):
    a. How do you choose a website or link to follow for more information?
    b. What features on a website are helpful?
    c. What features on a website are a hinderance?
    d. What puts you off looking at a certain website?

vi. Have you had any challenges accessing government services via online portals (e.g. MyGov)?

vii. Overall, what emotions best describe these experiences?

Q2. Can you tell me about your best experience of using the internet to access healthcare, information, and/or support?

Prompts:

i. Who was involved?
ii. Were there any thoughts that stood out to you?
iii. Overall, how did this experience make you feel?

Q3. Can you tell me about an experience of using the internet to access healthcare, information, and/or support that did not go so well?

Prompts:

i. Who was involved?
ii. Were there any thoughts or feelings that stood out to you?
iii. Was there something you expected to happen that did not happen?
iv. What do you think should have happened?
v. Is there something that would have made a difference?
vi. Overall, how did this experience make you feel?

Q4. Is there anything else you would like to add that I did not ask?

Emotion Prompt:

Select one (1) **emotion** most connected with experience:

Sad	Happy
Frustrated	Grateful
Anger	Content
Stressed	Satisfied
Confused	Hopeful
Left Out	Include

Other

Focus Group Guide: Family Members & Significant Others

Q1. Can you tell me about your experience(s) of supporting your [loved one/PWA/family member] to access health care, information, and support on the Internet?

Prompts:

i. Think back to the last time that you supported your loved one to use the Internet to access health care information or support. Tell me about that experience.
   a. Which site was it?
   b. Why were they going to that site?
   c. What happened when they tried to use the site?
   d. How often would they need to use that website?
   e. How did that make you feel? How do you think that made your [loved one/PWA/family member] feel?

ii. What kind of support did you provide your [loved one/PWA/family member]?
   a. How did you provide support?
   b. When did you provide support?

iii. Problem-solving & trouble-shooting on the Internet:
   a. Have there been times where your [loved one/PWA/family member] had challenges knowing what to search for?
      i. Did you help? How did you help?
   b. Has your loved one experienced challenges knowing what words to enter in their search?
      i. How have you provided support when they experience this?
   c. What do you do if there are words you or your [loved one/PWA/family member] do not recognise?

iv. Accessible Websites (Aphasia-friendly):
   a. In your experience, are health care website accessible for individuals with post-stroke?
   b. How do you select a website or link to follow for more information, that your [loved one/PWA/family member] can also access?
   c. What features on a website are helpful?
   d. What features on a website are a hinderance?
   e. What puts you or your [loved one/PWA/family member] off looking at a certain website?

v. Have you had any challenges accessing government services via online portals (e.g. MyGov)?

vi. Overall, what emotions best describe these experiences?

Q2. Can you tell me about your best experience of supporting your [loved one/PWA/family member] to access health care, information, and support on the Internet?

Prompts:

i. Who was involved?

ii. Were there any thoughts or feelings that stood out to you?

iii. Overall, how did this experience make you feel?

Q3. Can you tell me about an experience of supporting your [loved one/PWA/family member] to access health care, information, and support that did not go so well?

Prompts:

i. Who was involved?
ii. Were there any thoughts or feelings that stood out to you?
iii. Is there something that would have made a difference?
iv. Overall, how did this experience make you feel?

Q4. Is there anything else you would like to add that I did not ask?

Focus Group Guide: Health Professionals

Q1. Can you tell me about your experience(s) of helping patients with aphasia to use the Internet to access health care, information, and/or support on the Internet?

Prompts:

i. Think back to the last time that you supported a person with aphasia to use the Internet to access health care information or support. Tell me about that experience.
   a. Which site was it?
   b. Why did they need to access that site?
   c. How did they know about that site?
   d. What happened when they tried to use the site?
   e. How often would they need to use that website?
   f. Would there be times when they would need to use that website independently?
   g. How were you able to provide support in that situation?

ii. What has been your role in providing support?

iii. How have you helped/how do you help?

iv. What issues do your patients experience/tell you about?

v. In your experience, are healthcare websites aphasia-friendly?
   a. How are they/aren't they?
   b. What features help?
   c. What features are a hinderance?
   d. Aphasia formatting specifics:
      i. How do you know which key words to bold?
      ii. How do you know what visuals to select?
      iii. How do you decide which photos should be included? What visuals are best?

Q2. What has been your best experience of supporting a patient with aphasia to access health care, information, and support on the Internet?

i. Who was involved?

ii. How did this experience make you feel?

iii. Were there any thoughts or feelings that stood out to you?

iv. Did your experience differ from what you expected to happen?

Q3. Can you describe an experience of supporting a patient with aphasia to access health, information, and support on the Internet that did not go so well?

Prompts:

i. Is there something you would do differently?

ii. How did this experience make you feel?

iii. Are there any thoughts or feelings that stand out to you?

iv. Is there something that would make a difference?

Q4. Is there anything else you would like to add that I did not ask?

Demographic Information

People with Aphasia

- Age
- Gender
  - Male
  - Female
  - Another Term [please specify]
- Living region:
  - Metropolitan
  - Regional/remote
- Living situation
  - Living alone
  - Living with family
  - Living with professional carer
  - Other (please specify)
- Time post-onset of aphasia (dd/mm/yy)

Family Member/Carer/Significant Other

- Age
- Gender
  - Male
  - Female
  - Another Term [please specify]
- Relationship to person with aphasia:
  - Family member [please specify: spouse, child, parent, sibling, other]
  - Friend/Significant Other
  - Carer [professional, volunteer]
- Living region:
  - Metropolitan
  - Regional/remote
- Living situation:
  - Living with person with aphasia
  - Living separately to person with aphasia
  - Other (please specify)
- Time post-onset of family member's/client's/significant other's aphasia (dd/mm/yy)

Health Professionals

- Age
- Gender
  - Male
  - Female
  - Another Term [please specify]
- Clinical setting
  - Inpatient rehabilitation
  - Outpatient/community rehabilitation
  - Other [please specify]
- Work region
  - Metropolitan
  - Regional/remote
  - Other [please specify e.g. provide telehealth outreach from metropolitan base]
- Experience in providing aphasia services
  - < 1 year
  - 1-5 years
  - 5-10 years
  - > 10 years
