# Supplementary material for: Bridging the digital divide for people with aphasia: a study protocol for codesigning web accessibility tools and guidelines
Source: BMJ Open. 2025 Aug 10;15(8):e099273. doi: 10.1136/bmjopen-2025-099273 (PMC12336571; doi:10.1136/bmjopen-2025-099273)
Supplement: Supplementary file 2 [file bmjopen-2025-099273.reviewer_comments.pdf]

PEER REVIEW HISTORY

BMJ Open publishes all reviews undertaken for accepted manuscripts. Reviewers are asked to complete a checklist review form and are provided with free text boxes to elaborate on their assessment. These free text comments are reproduced below.

ARTICLE DETAILS

Title (Provisional)

Bridging the digital divide for people with aphasia: A study protocol for co-designing web accessibility tools and guidelines

Authors

Lee, Jennifer; Worthy, Peter; Deslandes, Ryan; Burton, Bridget; Copland, David A.; Jamieson, Phill; Barron, Kim; Togher, Leanne; Shrubsole, Kirstine; Shiggins, Ciara; Campbell, Jessica; Hill, Annie; Wiles, Janet; Haslam, Alex; Wallace, Sarah J. Jane

---

VERSION 1 - REVIEW

**Reviewer**	**1**
**Name**	**Menger, Fiona**
**Affiliation**	**Newcastle University, Speech and Language Sciences**
**Date**	**07-Mar-2025**
**COI**	**None**

Thank you for asking me to review this well-written and informative paper, which carefully describes the protocol for a much-needed piece of work to provide bespoke support for people with aphasia to access online information. This was something I discussed as a possibility for further research in the final chapter of my PhD and have long thought was needed. So I commend the team for designing this study, which centres the experiences and needs of people with aphasia in work that aims to find practical solutions to the ongoing challenges of digital exclusion. I find the rationale and methods sound and have only a few requests for clarifications/suggested amendments, plus some recommendations for published works the authors may find useful. See below:

• I am interested to know whether the proposed browser extension would work across platforms and devices, e.g. a desktop pc vs a mobile phone browser. Also, whether the funding includes maintenance and updates. The authors will no doubt be aware of the concerns that accessibility supports often cease to operate effectively as companies roll out software updates.

• What technological/IT/co-design expertise will be present across the stages?

• P7 line 121 – is it worth explicitly stating that the paper cited does not include those with acquired conditions?

• P8 line 138. Would this point in the paper be a good place to discuss digital exclusion in the wider population of Australia? It is important to recognise social as well as disability context.

• P9 line 39 – it may be worth expanding on what is meant by a recommender system. There may be concerns from participants about the extension collecting personal data to make recommendations?

• P11 Stage 1a of the table – clarify whether the aim is restricted to healthcare information or extends wider. Stage 1b aim 3 – does this need examples? Or expansion in the main text?

• P194 line 200 could be read as four family members of one person with aphasia.

• P13 line 228 – learning theory is mentioned without explanation – readers may benefit from a definition.

• P15 line 267 – authors may need to refer forward here to where criteria for participants is described.

• P15 line 274 –will you classify aphasia severity or is the review of severity aimed at facilitating communication?

• P15 line 287 – Talking Mats needs a trademark.

• P15 section on supported communication. I'm interested as to whether you will use anything such as mock up layouts to support stage 1b. See references provided below.

• P16 line 39. Can you elaborate on 'touchpoint materials'?

• P17 – No heading provided for healthcare professionals. This section may need greater synthesis around how people with aphasia and family members will be approached then recruited. If a couple both express interest, would you meet and consent them together?

• P19 line 361. Would you need to collect separate consent for recording of the groups?

• P19 line 366 onwards. You do not include proxy use here, although may be considering that under 'providing support'. It is important to make the distinction between supported use and proxy use.

• P20 line 400 – could you give some examples here, e.g. physical ranking/sorting of priorities?

• P22 Discussion – this is not really a discussion section, more a summary.

• The following research may already be known to the team. It has relevance as there is some precedent for co-design with people with aphasia.

o Galliers, J., Wilson, S., Roper, A., Cocks, N., Marshall, J., Muscroft, S., & Pring, T. (2012, August). Words are not enough: empowering people with aphasia in the design process. In

Proceedings of the 12th Participatory Design Conference: Research Papers-Volume 1 (pp. 51-60).

o Wilson, S., Roper, A., Marshall, J., Galliers, J., Devane, N., Booth, T., & Woolf, C. (2015). Codesign for people with aphasia through tangible design languages. CoDesign, 11(1), 21-34.

o Boyd-Graber, J. L., Nikolova, S. S., Moffatt, K. A., Kin, K. C., Lee, J. Y., Mackey, L. W., ... & Klawe, M. M. (2006, April). Participatory design with proxies: developing a desktop-PDA system to support people with aphasia. In Proceedings of the SIGCHI conference on Human Factors in computing systems (pp. 151-160).

o Buhr, H. R., Hoepner, J. K., Miller, H., & Johnson, C. (2016). AphasiaWeb: development and evaluation of an aphasia-friendly social networking application. Aphasiology, 31(9), 999–1020. https://doi.org/10.1080/02687038.2016.1232361

o Language-Light UX Guidelines for Aphasia for designers of software, apps, and websites. https://languagelightux.org/

Fiona Menger

7 March 2025

**Reviewer**	**2**
**Name**	**Cocks, Naomi**
**Affiliation**	**Curtin University**
**Date**	**26-Mar-2025**
**COI**	**None**

Thank you for asking me to review this article which describes a protocol for a study in which a web browser extension will be developed for people with aphasia.

The study is well-designed and I look forward to reading the outcomes of the study.

While the paper reads well, the protocol is not particularly novel or new. The authors describe a process in which there are co-design groups with people with aphasia, health professionals and family members. This is a well-established approach to this kind of research. I would like to see more of a discussion about how this protocol is novel and new. I would like to see a bit more of a justification as to why publishing this protocol will be of interest to readers. It was also not a very complex protocol and so I wasn't sure such a detailed paper just on the protocol was needed. I was wondering once I got to the end of reading it why there was a need to publish the protocol and why I couldn't just have read the publications at the end of each of the stages or one large final publication. If the authors can more clearly justify why it would be important to publish this protocol rather than just

publishing the methods as parts of research papers, then I think the paper should be published.

Some more detailed feedback is as follows:

Line 40 Methods and analysis- “Experience-based co-design” is not a full sentence. Is it meant to be a subheading? I think avoid sub-headings in abstract

Line 74- Background- first sentence that begins “communication is central” has lots of parts and is difficult to follow. Please break into multiple sentences.

Line 82- the list of causes to damage to the brain is not complete. There are many more causes. Instead just put “including..” before the list so that it is clear this is not the entire list of possible causes.

Line 84- not all people who have had a stroke like to be referred to as “survivors’ consider rewording to avoid this word.

Line 88- again remove the word “survivors”.

Line 264- this seems a very short exclusion and inclusion criteria for both. Are you including people with cognitive impairment? What about visual and hearing loss? What about other neurological diagnoses? What about ability to use upper limbs? Etc etc Please consider all possible profiles of individuals.

Line 266- are you going to state a minimum number of years working with people with aphasia or percentage of caseload of people who have aphasia for the health professionals to ensure they are sufficiently knowledgeable?

Line 452- who will build the prototype? What is their experience of aphasia?

VERSION 1 - AUTHOR RESPONSE

Reviewer 1

Dear Dr Menger,

Thank you very much for your encouraging and positive feedback on our manuscript. We are grateful for this opportunity to follow on from your body of research. Thank you also for the recommended readings.

Reviewer comments	Authors response	Changes in manuscript
I am interested to know whether the proposed browser extension would work across platforms and devices, e.g. a	The browser extension in this study will be developed for use on a computer only, including desktop PCs and laptops. There are additional	Page 21, Line 483<br>Page 24, Line 570

desktop pc vs a mobile phone browser. Also, whether the funding includes maintenance and updates. The authors will no doubt be aware of the concerns that accessibility supports often cease to operate effectively as companies roll out software updates.	considerations and development challenges for creating browser extensions for mobile phone and tablet browsers. However, given that more people are connecting via their phones or tablets nowadays, this is something that may be investigated in a future research project. We have clarified this in the aims and included a brief discussion of this in the summary section of the manuscript.<br><br>The funding awarded is for co-designing, developing, and testing for the browser extension prototypes. Throughout the project, the researchers and co-designers are considering sustainability.	
What technological/IT/co-design expertise will be present across the stages?	Expertise in technology design/IT and co-design will be present across all stages of the project. We have highlight some of this expertise below and in the text:<br><br>KB and PJ are both chief investigators on the project and have lived experience with post-stroke aphasia. They both work in the Tech Hub at our research centre and have worked on several tech co-design projects for people with aphasia, including working with Amazon Web Services. KB has a background in user experience design and PJ has a background in customer experience.<br><br>PW (interaction designer) has expertise in co-designing and developing technology for and with a wide range of peoples (people living with dementia, young children with developmental challenges, people living with chronic pain, first nation's men supporting their mental wellbeing).	Page 19, Line 432<br>Page 21, Line 480

	RD (software developer) has experience with developing technology for people living with dementia and has worked on a number of projects with people living with aphasia.<br><br>SJW is a speech pathology with extensive experience in co-design and will oversee the project.	
P7 line 121 – is it worth explicitly stating that the paper cited does not include those with acquired conditions?	Thank you for this suggestion. We have now added a statement to highlight this.	Page 5, Line 112
P8 line 138. Would this point in the paper be a good place to discuss digital exclusion in the wider population of Australia? It is important to recognise social as well as disability context.	Thank you also for this suggestion. We agree that it is important to understand the social context of digital exclusion in Australia, and have included a brief discussion of this.	Page 5, Line 105
P9 line 39 – it may be worth expanding on what is meant by a recommender system. There may be concerns from participants about the extension collecting personal data to make recommendations?	Yes, the recommender system may need to have access to a user's browsing history to make further suggestions. Considerations for secure storage of personal data and browsing data will need to be discussed in the co-design workshops.	Page 20, Line 468
P11 Stage 1a of the table – clarify whether the aim is restricted to healthcare information or extends wider. Stage 1b aim 3 – does this need examples? Or expansion in the main text?	Stage 1a aim: We will include open ended questions to capture internet use more broadly and some specific questions that target health and government access. This has been clarified in the manuscript. The discussion guide has also been included in Supplementary materials.<br><br>Stage 1b aim 3: An example of potential future developments may be browser extensions that are compatible with mobile devices or other app-based technologies.	Page 8, Table 1
P14 line 200 could be read as four family members of one person with aphasia.	Thank you for pointing out the ambiguity of this sentence. We have amended the description to: "four	Page 9, Line 183

	unrelated family members of people with post-stroke aphasia”	
P13 line 228 – learning theory is mentioned without explanation – readers may benefit from a definition.	We have included a short description of learning theory in the context of co-design.	Page 10, Line 210
P15 line 267 – authors may need to refer forward here to where criteria for participants is described.	Thank you for this suggestion. We have now included a statement that directs the reader to the ‘Sampling’ section for more details on inclusion and exclusion criteria.	Page 12, Line 252
P15 line 274 –will you classify aphasia severity or is the review of severity aimed at facilitating communication?	Yes, aphasia severity will be a consideration as part of sampling matrix. The review of severity may also be used to understand communication support needs during the focus groups/workshops. Participants will also be asked to describe their communication preferences in the initial meeting.	No changes made
P15 line 287 – Talking Mats needs a trademark.	The trademark has been added	Page 12, Line 274
P15 section on supported communication. I’m interested as to whether you will use anything such as mock up layouts to support stage 1b. See references provided below.	Thank you very much for the reading recommendations. Yes, we will use mock-up layouts during the co-design workshops. These may be created collaboratively using Miro or co-designers may draw mock-ups in their design journal and share them with us. We have included more details on materials that will be used in the co-design stage.	Page 20, Line 460
P16 line 39. Can you elaborate on ‘touchpoint materials’?	We have provided examples of potential touchpoint materials that will be created as part of the project.	Page 13, Line 288
P17 – No heading provided for healthcare professionals. This section may need greater synthesis around how people with aphasia and family members will be approached then recruited. If a couple both express interest, would you meet and consent them together?	Thank you for this point of clarification. We have revised the manuscript to include a heading for each stakeholder group. Under ‘Family members or significant others’, we have included the process that will be taken if a family member expresses interest as an individual or with their family member with aphasia.	Page 14, Line 318

P19 line 361. Would you need to collect separate consent for recording of the groups?	Participants will be asked to provide consent to being recorded during the initial consent process by acknowledging this on the consent form. At the start of each meeting/focus group/workshop, the researcher will confirm consent by asking participants if they are happy to be recorded and by monitoring responses (e.g. nodding, thumbs up, saying yes). We have included this in the manuscript.	Page 16, Line 357
P19 line 366 onwards. You do not include proxy use here, although may be considering that under 'providing support'. It is important to make the distinction between supported use and proxy use.	Thank you highlighting this important distinction between communication support and proxy communication. We have included more detail about the primary role of the support person in the focus group, which is to provide communication support. However, we acknowledge that there may be instances where a support person may act as a communication proxy and have noted this in the text. We have described ways to verify that responses reflect the participant with aphasia's experiences.	Page 17, Line 378
P20 line 400 – could you give some examples here, e.g. physical ranking/sorting of priorities?	This activity will be completed using the Miro board. More details have been provided in the manuscript.	Page 18, Line 405
P22 Discussion – this is not really a discussion section, more a summary.	We agree that this section reads more like a summary than a discussion, thus have changed the heading of the section to 'Summary'. We have also added some foreseeable limitations to this section.	Page 23, Line 541<br>Page 24, 571

Reviewer 2

Dear Dr Cocks,

Thank you for your thoughtful comments on our manuscript and for highlighting areas that need to be better articulated.

We acknowledge that co-design methodologies involving people with aphasia, health professionals, and family members are established approaches. The specific use of EBCD in development of a web browser extension for people with aphasia is novel. The combination of EBCD and human-centred design (HCD) to design technology for people with aphasia is also a novel approach. Further, we respectfully suggest that the value of a protocol paper extends beyond presenting novel methods alone. We believe publishing this protocol serves several important functions:

- While co-design approaches are well-established, our application of EBCD to aphasia tech development involves important adaptations and considerations that may benefit others working in similar contexts.
- Bringing together EBCD and HCD extends EBCD into technology design. It also provides a useful methodology for people using HCD in tech design and development to highlight lived experiences and key touchpoints.
- Documentation of strategies to facilitate co-design activities online.
- Technology development occurs rapidly. Publishing our protocol allows other researchers to be aware of our ongoing work, potentially reducing unnecessary duplication of effort and facilitating collaboration.
- Publishing a detailed protocol before study completion enhances methodological transparency and enables other researchers to replicate or adapt our approach.

While future papers will certainly report on methods, space constraints often limit the detail that can be provided, particularly regarding the nuanced decision-making processes involved in co-design with people with communication difficulties. Publishing this protocol allows us to document these considerations more comprehensively than would be possible in standard research papers. We have revised the manuscript (see Page 6, Lines 141 - 151) to more explicitly address these points and better articulate the value of publishing this protocol.

**Reviewer comments**	**Author responses**	**Changes in manuscript**
Line 40 Methods and analysis- “Experience-based co-design” is not a full sentence. Is it meant to be a subheading? I think avoid sub-headings in abstract	Thank you for this suggestion. We have reworded this into a sentence.	Page 2, Line 40
Line 74- Background- first sentence that begins “communication is central” has lots of parts and is difficult to follow. Please break into multiple sentences.	Thank you for pointing this out. We have revised this sentence to enhance its readability. The introduction has also been edited to enhance readability.	Page 4, Line 73
Line 82- the list of causes to damage to the brain is not complete. There are many more causes. Instead just put	We have revised this sentence as suggested.	Page 4, Line 79

"including.." before the list so that it is clear this is not the entire list of possible causes.		
Line 84- not all people who have had a stroke like to be referred to as "survivors' consider rewording to avoid this word.	Thank you for highlighting this very important point. We have removed 'survivors' and reworded this sentence.	Page 4, Line 81
Line 88- again remove the word "survivors".	Likewise, we have removed 'survivors' and reworded this sentence. We have also carefully reviewed the manuscript to ensure that this term is not used.	Page 4, Line 95
Line 264- this seems a very short exclusion and inclusion criteria for both. Are you including people with cognitive impairment? What about visual and hearing loss? What about other neurological diagnoses? What about ability to use upper limbs? Etc etc Please consider all possible profiles of individuals.	Thank you for pointing out other considerations for our eligibility criteria. Our selection criteria aims to be as inclusive as possible. However, we have added people with severe cognitive impairments that would hinder their ability to provide informed consent, people with neurodegenerative conditions, and people with aphasia not caused by stroke to the exclusion criteria.	Page 11, Line 243
Line 266- are you going to state a minimum number of years working with people with aphasia or percentage of caseload of people who have aphasia for the health professionals to ensure they are sufficiently knowledgeable?	To be inclusive of all health professionals, we will not state a minimum number of years working with people with aphasia. However, in our sampling of health professionals, we will aim to recruit clinicians with a range of experiences (e.g. years of experience, clinical setting)	No changes made, but please see Sampling section on Page 12, Line 254
Line 452- who will build the prototype? What is their experience of aphasia?	Authors PW (interaction designer) and RD (software developer) will be building the prototypes. PW has expertise in co-designing and developing technology for and with a wide range of peoples (people living with dementia, young children with developmental challenges, people living with chronic pain, first nation's men supporting their mental wellbeing). RD has experience in developing technology for people with aphasia and has worked on a number of	Page 21, Line 481

	projects with people living with aphasia. These details have been added into the manuscript.	

VERSION 2 - REVIEW

Reviewer 1
Name Menger, Fiona
Affiliation Newcastle University, Speech and Language Sciences
Date 06-Jun-2025
COI

Thank you for a further opportunity to look at this paper. I am satisfied that the authors have fully responded to the comments of both reviews, which represent different perspectives in critically evaluating the work. The paper is improved as a result and I would recommend publication. I have two very minor points.

1. The authors have included information on the context of digital inclusion in Australia. P5 line 105. Should the Thomas et al. citation be moved to after the point on groups most impacted, or is this information from a separate source?

2. I think we have slightly crossed wires when discussing proxy internet use. I was referring to proxy use as discussed in the paper below, which you already cite. Apologies that this was not clear! The data is older now, but we found that people with and without aphasia post stroke commonly used the Internet via a proxy, e.g., someone else would look up information, then pass it on in an accessible way. Many were happy with that arrangement, which is of course dependent on the availability of the proxy… What I was trying to convey was the need to acknowledge a spectrum of digital independence (before and after stroke), which may vary by task complexity (e.g., form filling more demanding than accessing news) and that independent use across all contexts may not be a desired goal for all. Those involved in your research are perhaps more likely to be those motivated for more independent use. My point was to acknowledge the difference between direct support/facilitation (sitting side by side someone online) and searching for information or completing tasks by proxy.

Menger, F., Morris, J., & Salis, C. (2020). The impact of aphasia on Internet and technology use. Disability and rehabilitation, 42(21), 2986-2996.

Your interpretation around communication support was also an important clarification!

**Reviewer**	**2**
**Name**	**Cocks, Naomi**
**Affiliation**	**Curtin University**
**Date**	**29-May-2025**
**COI**	

The rationale for publishing this protocol has been articulated more clearly, effectively highlighting the specific gap it addresses within the existing body of research. All other reviewer comments have been thoroughly and appropriately addressed.

VERSION 2 - AUTHOR RESPONSE

Reviewer 1

Dear Dr. Menger,

Thank you for your time in reviewing our revised manuscript and providing us with thorough feedback. We have addressed your comments below, and hope that this will help improve the clarity of the manuscript.

Reviewer comments	Authors response	Changes in manuscript
The authors have included information on the context of digital inclusion in Australia. P5 line 105. Should the Thomas et al. citation be moved to after the point on groups most impacted, or is this information from a separate source?	Thank you for highlighting this missing references.<br>We have now included the Thomas et al. citation to the sentence that lists the groups most impacted.	Page 5, Line 109
I think we have slightly crossed wires when discussing proxy internet use. I was referring to proxy use as discussed in the paper below, which you already cite. Apologies that this was not clear! The data is older now, but we found that people with and without aphasia post stroke commonly used the Internet via a proxy, e.g., someone else would look up information, then pass it on in an accessible way. Many	Apologies that we have misinterpreted this point and thank you for the clarification. We have included a brief description for how we will capture the range of digital independence in our participants and have acknowledged that some users may prefer proxy internet use.	Page 16, Line 371

were happy with that arrangement, which is of course dependent on the availability of the proxy… What I was trying to convey was the need to acknowledge a spectrum of digital independence (before and after stroke), which may vary by task complexity (e.g., form filling more demanding than accessing news) and that independent use across all contexts may not be a desired goal for all. Those involved in your research are perhaps more likely to be those motivated for more independent use. My point was to acknowledge the difference between direct support/facilitation (sitting side by side someone online) and searching for information or completing tasks by proxy.<br><br>Menger, F., Morris, J., & Salis, C. (2020). The impact of aphasia on Internet and technology use. Disability and rehabilitation, 42(21), 2986-2996.<br><br>Your interpretation around communication support was also an important clarification!		
